# Crowdsourcing in health and medical research: a systematic review

**DOI:** 10.1186/s40249-020-0622-9

**Published:** 2020-01-20

**Authors:** Cheng Wang, Larry Han, Gabriella Stein, Suzanne Day, Cedric Bien-Gund, Allison Mathews, Jason J. Ong, Pei-Zhen Zhao, Shu-Fang Wei, Jennifer Walker, Roger Chou, Amy Lee, Angela Chen, Barry Bayus, Joseph D. Tucker

**Affiliations:** 1 Dermatology Hospital of Southern Medical University, Guangzhou, China; 2 Guangdong Provincial Center for Skin Diseases and Sexually Transmitted Infection Control, Guangzhou, China; 3 Department of Biostatistics, Harvard Chan School of Public Health, Boston, MA USA; 4 Social Entrepreneurship to Spur Health, Guangzhou, China; 5 Institute of Global Health and Infectious Diseases, University of North Carolina at Chapel Hill, Chapel Hill, USA; 6 Department of Social Medicine, University of North Carolina School of Medicine, Chapel Hill, USA; 7 University of North Carolina Project-China, Guangzhou, China; 8 Faculty of Infectious and Tropical Diseases, London School of Hygiene and Tropical Medicine, London, UK; 9 Health Sciences Library, University of North Carolina, Chapel Hill, USA; 10 Department of Medical Informatics and Clinical Epidemiology, Oregon Health & Sciences University, Portland, USA; 11 Department of Medicine, Oregon Health & Sciences University, Portland, USA; 12 Department of Orthopaedics, Brigham and Women’s Hospital, Boston, USA; 13 Kenan-Flagler School of Business, University of North Carolina at Chapel Hill, Chapel Hill, USA

**Keywords:** Crowdsourcing, Innovation, Challenge contest, Systematic review, Medicine, Health

## Abstract

**Background:**

Crowdsourcing is used increasingly in health and medical research. Crowdsourcing is the process of aggregating crowd wisdom to solve a problem. The purpose of this systematic review is to summarize quantitative evidence on crowdsourcing to improve health.

**Methods:**

We followed Cochrane systematic review guidance and systematically searched seven databases up to September 4th 2019. Studies were included if they reported on crowdsourcing and related to health or medicine. Studies were excluded if recruitment was the only use of crowdsourcing. We determined the level of evidence associated with review findings using the GRADE approach.

**Results:**

We screened 3508 citations, accessed 362 articles, and included 188 studies. Ninety-six studies examined effectiveness, 127 examined feasibility, and 37 examined cost. The most common purposes were to evaluate surgical skills (17 studies), to create sexual health messages (seven studies), and to provide layperson cardio-pulmonary resuscitation (CPR) out-of-hospital (six studies). Seventeen observational studies used crowdsourcing to evaluate surgical skills, finding that crowdsourcing evaluation was as effective as expert evaluation (low quality). Four studies used a challenge contest to solicit human immunodeficiency virus (HIV) testing promotion materials and increase HIV testing rates (moderate quality), and two of the four studies found this approach saved money. Three studies suggested that an interactive technology system increased rates of layperson initiated CPR out-of-hospital (moderate quality). However, studies analyzing crowdsourcing to evaluate surgical skills and layperson-initiated CPR were only from high-income countries. Five studies examined crowdsourcing to inform artificial intelligence projects, most often related to annotation of medical data. Crowdsourcing was evaluated using different outcomes, limiting the extent to which studies could be pooled.

**Conclusions:**

Crowdsourcing has been used to improve health in many settings. Although crowdsourcing is effective at improving behavioral outcomes, more research is needed to understand effects on clinical outcomes and costs. More research is needed on crowdsourcing as a tool to develop artificial intelligence systems in medicine.

**Trial registration:**

PROSPERO: CRD42017052835. December 27, 2016.

## Background

Conventional, expert-driven solutions to medical problems often fail. Innovative approaches such as crowdsourcing may provide a useful community-based method to improve medical services. Crowdsourcing is the process of aggregating crowd wisdom in order to solve a problem [1]. This involves a group solving a problem and then sharing the solution. For example, the initiation of out-of-hospital cardiopulmonary resuscitation (CPR) is often delayed, leading to considerable morbidity and mortality. To address this problem, several teams organized a crowdsourced solution — [2–7] training lay people to administer out-of-hospital CPR. When emergency medical services received a call, they sent a text message to proximate laypeople who then provided CPR. This system has been formally evaluated in several studies [3, 4].

Crowdsourcing approaches are increasingly used in public health and medicine [8, 9]. Examples include engaging youth in developing HIV services [10], designing a patient-centered mammography report [11], and enhancing cancer research [12]. Some crowdsourcing approaches focus on the process of mass community engagement, obtaining creative input from many individuals [13, 14]. Other work has focused on the collective input of participants to generate a single, high-quality output such as clinical algorithms [15–18]. The crowd in crowdsourcing may be members of the general public [19] or individuals with specific clinical expertise [20]. Recognizing the growing importance of crowdsourcing, the United Nations International Children’s Emergency Fund (UNICEF)/ The United Nations Development Programme (UNDP)/World Bank/ The World Health Organisation (WHO) Special Programme for Research and Training in Tropical Diseases (TDR) published a practical guide on crowdsourcing in health and health research [21].

Despite the growth of crowdsourcing in medical settings, few systematic reviews have focused on evaluating crowdsourcing research in medicine [18, 22]. To date, existing reviews have been general [22], have largely ignored crowdsourcing in medicine [9, 18], and have not incorporated the most recent literature [9, 22]. A systematic analysis of the expanding medical literature on crowdsourcing is needed to understand optimal methods. The purpose of this systematic review is to summarize quantitative evidence on crowdsourcing to improve health.

## Methods

### Search strategy

Based on the Preferred Reporting Items for Systematic Reviews and Meta-Analyses (PRISMA, http://www.prisma-statement.org/) checklist and Cochrane guidance, we searched the following seven databases: MEDLINE (via PubMed), Embase, CINAHL, Web of Science, PsycINFO, Cochrane, and ABI/Inform [23, 24]. The search algorithm included elements related to crowdsourcing and to health (Additional file 1: Tables S1–S7). Databases were initially searched on December 7, 2016 and updated on September 4th, 2019. Bibliographies of included articles were also hand searched to identify additional relevant studies.

Inclusion criteria were defined a priori in a protocol registered on PROSPERO, an international prospective register of systematic reviews (CRD42017052835: https://www.crd.york.ac.uk/prospero/display_record.php?RecordID=52835). Articles were included if they were peer-reviewed, reported on crowdsourcing, and were directly related to health. Studies had to report quantitative data on behavioral outcomes, clinical outcomes, feasibility, or cost. We included peer-reviewed research studies described in abstracts if associated original research manuscripts were not included. Exclusion criteria included: failure to provide sufficient detail of methods, use of crowdsourcing only for participant recruitment, qualitative study, non-English study, or non-empirical study. Studies using crowdsourcing to conduct systematic reviews were not included.

### Study selection

**Fig. 1:**
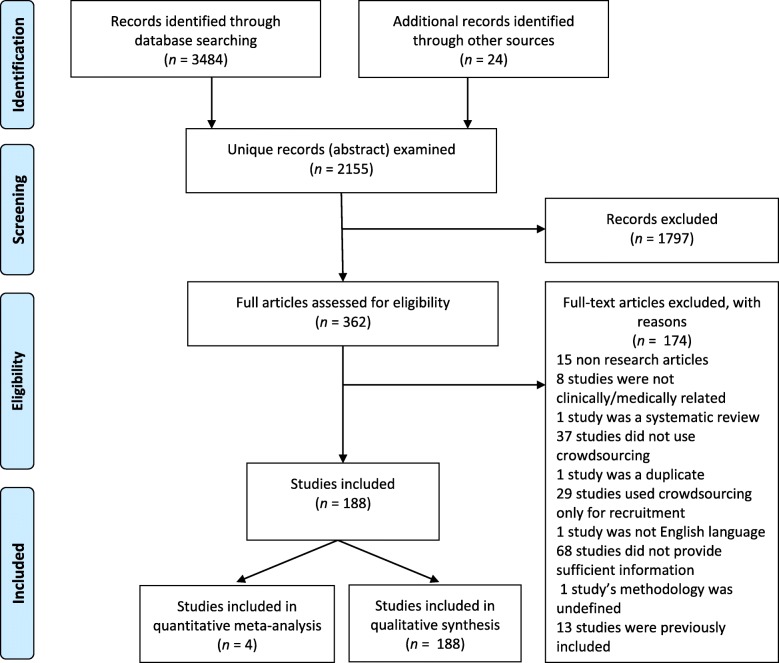
Overview of study selection data abstraction

After duplicates were removed, screening proceeded in two stages (Fig. 1). First, one individual reviewed the abstract and title of each article according to the criteria mentioned above. A full text review was then conducted with two to four individuals independently evaluating each article. Disagreements on whether to include a full text article were resolved by the senior author. Screening and data extraction occurred once for each selected study.

The following fields underwent dual extraction: citation information (first author, study year, PMID), study setting (nation, city), target health focus/condition, study design, purpose, number of contributions, and study findings. We collected data about effectiveness (focusing on behavioral and clinical outcomes), feasibility, and cost. Effectiveness data included studies that evaluated some health outcome. Feasibility studies examine the feasibility of implementing a crowdsourcing approach in a health context. Cost analysis data provided economic or financial costs associated with the crowdsourcing intervention. We pooled applicable data using meta-analysis if studies used a similar intervention and reported similar metrics. We used random effects models and analysis was undertaken using RevMan 5. Study heterogeneity was assessed by calculating I-squared values. We assessed for small sample size effects using funnel plots if there were more than ten studies.

### GRADE evidence profile

For each study, we examined the risk of bias tables, study limitations, consistency, precision, directness, and other factors described in the supplementary tables. Review findings were assessed as high, moderate, low, or very low, reflecting certainty in the estimates. We used the GRADE approach to assess the certainty of the summary finding. The GRADE evidence profile was compiled separately for observational studies and randomized controlled trials (RCTs) for surgical skills, sexual health messages, and out-of-hospital CPR.

We used the Cochrane Collaboration’s tool to assess risk of bias in RCT studies [25]. We used a separate tool to assess the risk of bias of observational studies [26]. Selection bias (development and application of eligibility criteria, controlled for confounding), detection bias (measurement of exposure and outcome), and attrition bias (follow-up) were assessed for each observational study of surgical skills, sexual health messages, and out-of-hospital CPR.

## Results

### Description of included studies

The database searches and selection of articles from references yielded 2155 unique citations. After screening abstracts, the full texts of 362 articles were reviewed. One hundred and seventy-four articles were excluded during full text screening: 15 were non-research articles; 37 did not use crowdsourcing; 13 contests were described in two papers each and we used the study that most comprehensively described the contest; 68 did not have enough information; 29 studies only used crowdsourcing for recruitment; one study was not in English; eight studies were not clinically/medically related; one study was a duplicate not previously excluded; one study was a systematic review; and one study’s methodology was unclear. One hundred and eighty-eight studies met the inclusion criteria and four studies were pooled (Fig. 1).

### Study characteristics

There were 183 observational studies and five RCTs. Nine studies were conducted in multiple countries, 166 studies were in high-income countries, 14 were in middle-income countries, and two were in low-income countries. Overall, 96 studies examined effectiveness, 127 examined feasibility, and 37 examined cost. Among those that examined effectiveness, all reported a behavioral outcome with the exception of two studies which reported a clinical outcome: measures of motor performance [27] and electrodermal activity [28].

### Synthesizing evidence

We examined data from studies that evaluated surgical skills (17 studies) [29–42], generated sexual health messages (seven studies) [13, 43–48], developed systems for out-of-hospital cardiopulmonary resuscitation (six studies) [2–7], quantified malaria parasitemia (two studies) [15, 49], and generated messages for smoking cessation (three studies) [50–52].

**Fig. 2:**
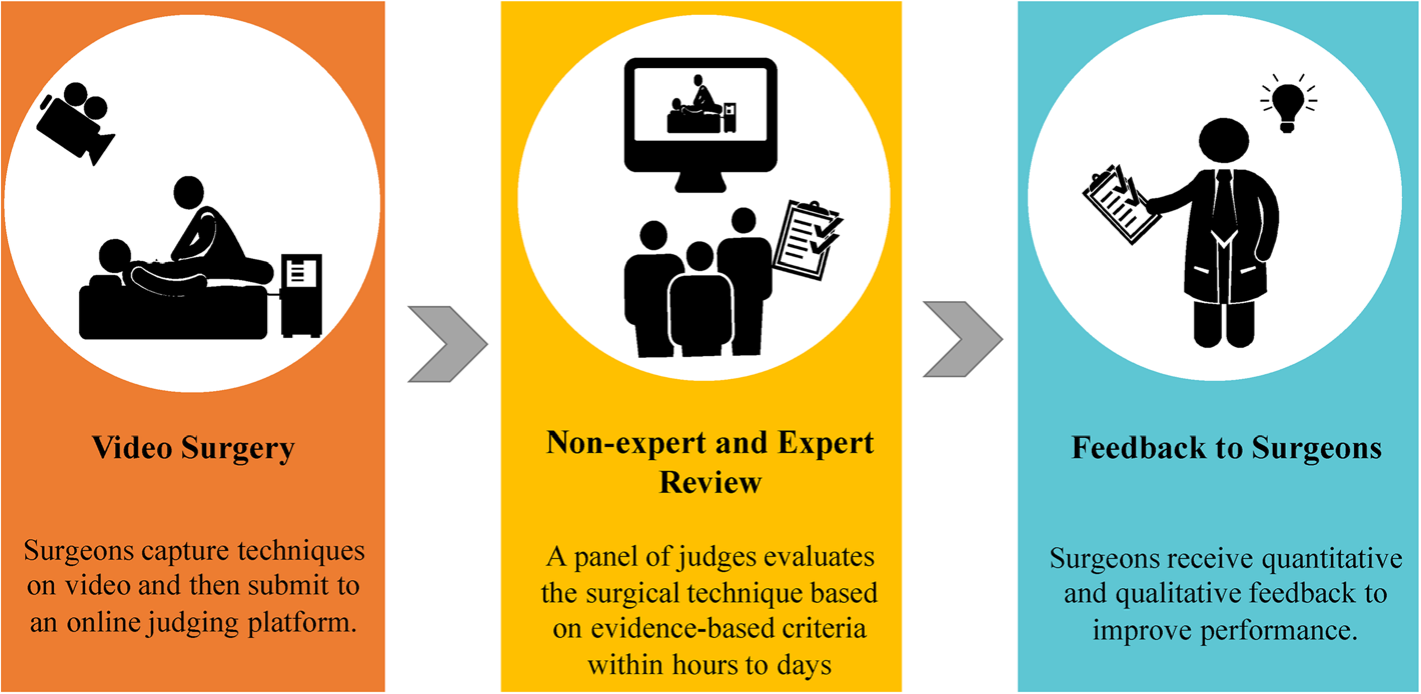
Process of using crowdsourcing to evaluate surgical performance

Of the 17 studies that used crowdsourcing to evaluate surgical skills, 16 found the crowdsourcing evaluations were effective compared to expert evaluations. Crowdsourcing evaluation typically involves videotaping a surgeon performing a skill in the surgical theatre and then uploading it onto a platform where an online crowd worker evaluates skill based on pre-specified criteria (Fig. 2). All 16 studies paid non-expert, online, crowd workers small amounts of money to evaluate surgical skills. Sixteen studies compared crowdsourcing approaches to conventional expert-panel approaches (see Additional file 2: Table S8, Additional file 3: Table S9, Additional file 6: Table S12). Low quality evidence from these studies suggested that crowd evaluation of surgical skill technique correlated with expert evaluation (see Additional file 3: Table S9). Moderate quality evidence suggested that crowdsourcing evaluation was faster than expert evaluation (see Additional file 3: Table S9). Due to the heterogeneity of measures, we were only able to pool data from two of these studies with similar interventions and measures, with the results suggesting no difference between crowdsourced and expert evaluation (*P* = 0.29) (see Additional file 4: Figure S10).

**Fig. 3:**
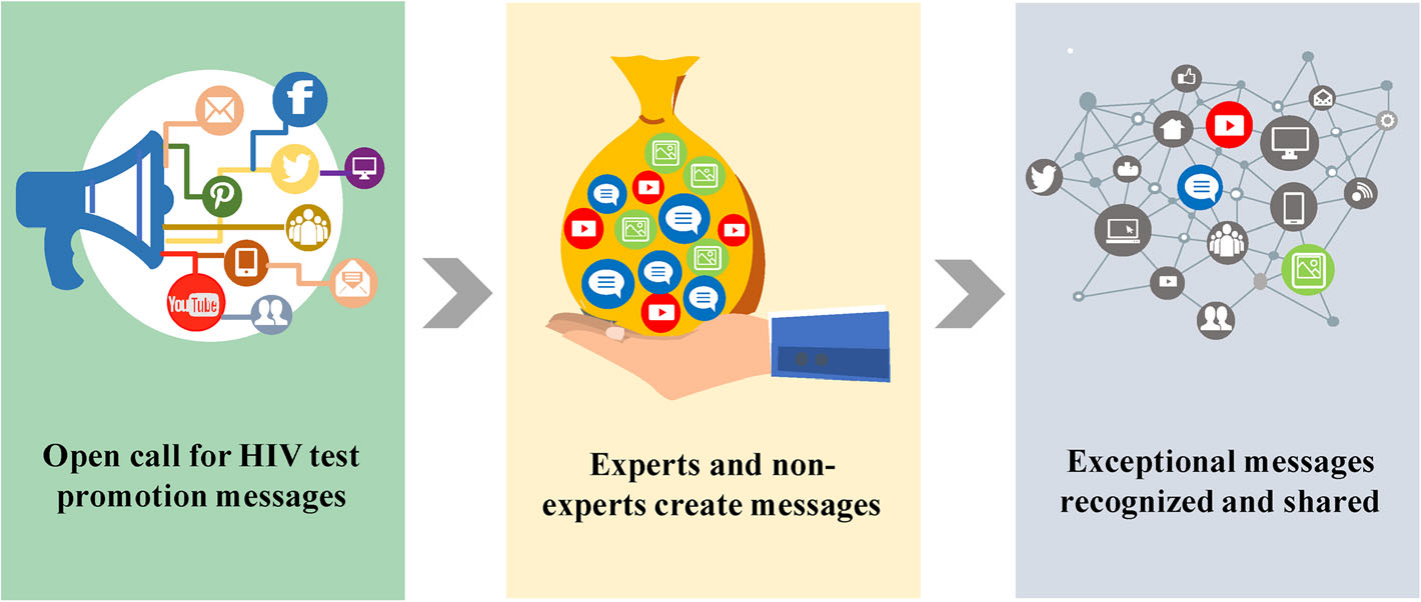
Process of using crowdsourcing to increase HIV testing

Seven studies evaluated innovation design contests to develop sexual health messages (Fig. 3, Additional file 5: Table S11, Additional file 6: Table S12) [13, 43–48]. Six of these studies were focused on low and middle income countries (LMICs) (Swaziland, Namibia, Kenya, Senegal, Burkina Faso, Nigeria, China) [13, 43, 45–48] and one was in a high-income country (United States) [44]. Both quantitative sexual health studies were designed as non-inferiority studies and found similar effectiveness when comparing crowdsourcing and social marketing approaches (see Additional file 4: Figure S10) [46, 48]. Both reported substantial cost savings associated with crowdsourcing compared to a conventional approach [46, 48]. There was moderate quality evidence from four studies (two RCTs, two observational studies) supporting innovation design contests to increase HIV testing (see Additional file 7: Table S13). There was moderate quality evidence from six studies (two RCTs, four observational studies) supporting innovation design contests to increase sexual health communication among youth (see Additional file 7: Table S13).

**Fig. 4:**
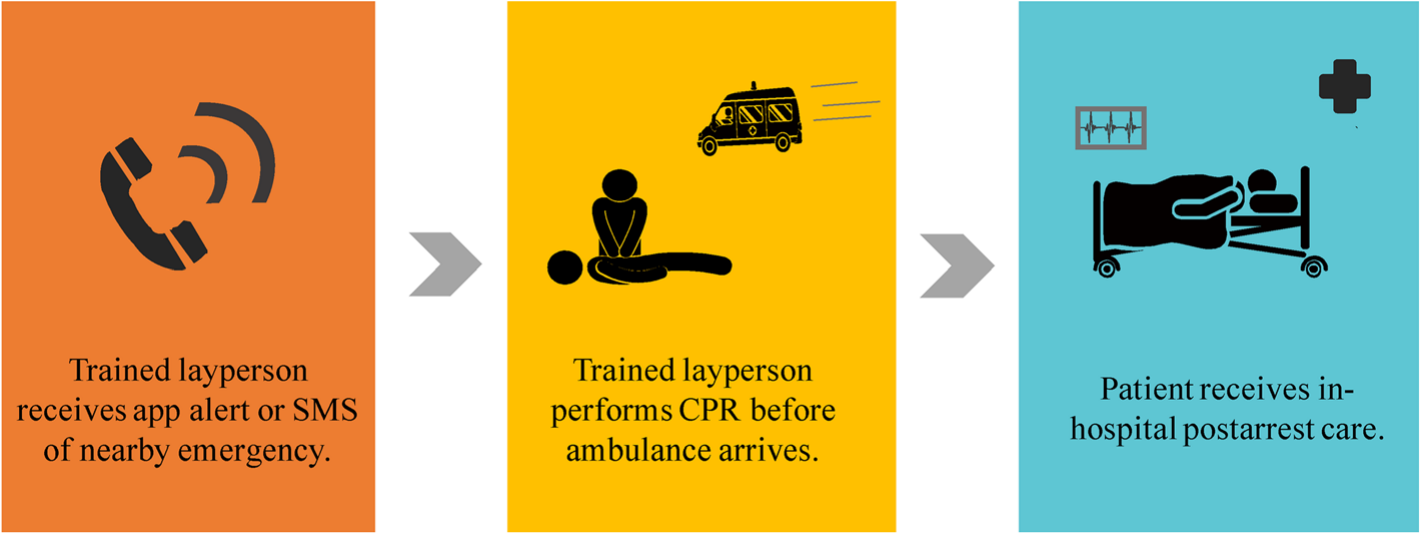
Process of using crowdsourcing to facilitate layperson CPR outside of the hospital. CPR: Cardiopulmonary resuscitation; SMS: Short message service

Six studies evaluated out-of-hospital layperson-facilitated CPR (Fig. 4, see Additional file 8: Table S14, Additional file 9: Table S15, Additional file 10: Table S16) [2–7]. Two were RCTs conducted in high-income European countries (Sweden, Germany) which showed that bystander-initiated CPR was more frequent in the intervention group (using the smartphone app) but not necessarily faster [5, 7]. The four observational studies were also conducted in high-income countries (US, Japan, Sweden, Netherlands) [2–4, 6] and indicated the feasibility of the use of smartphone apps and SMS to increase layperson-facilitated CPR. We found moderate evidence to support smartphone apps and SMS to increase out-of-hospital CPR while emergency responders are en route. The data on using crowdsourced systems to improve time to CPR is mixed. The one RCT that failed to find a difference between a crowdsourced intervention and a control group had potential bias [7].

Five studies used crowdsourcing to develop artificial intelligence projects [53–57]. Four of these studies annotated medical data to train machine learning algorithms [53, 55–57]. One study found that a three-phase crowdsourcing challenge contest could be used to develop an artificial intelligence algorithm to segment lung tumors for radiation therapy [54]. The best algorithms developed from this challenge contests were similar in effectiveness to human experts.

Among the three studies evaluating crowdsourcing to spur smoking cessation, one study found that this approach was not effective [50], and one study found an increase in smoking cessation after the contest [51]. For quantifying malaria parasitemia, crowdsourcing was found to be effective in both of two studies [15, 58]. Two studies found that crowdsourcing could be used to effectively identify malaria species [59, 60]. Two studies examined crowdsourcing to enhance identification of seizures, both finding that it was effective [61, 62].

## Discussion

Our systematic review identified crowdsourcing approaches using a variety of techniques and in different medical contexts. These data suggest crowdsourcing may be a useful tool in many settings. Evidence was most robust on crowdsourcing for evaluating surgical skills, increasing HIV testing, and organizing layperson assisted out-of-hospital CPR.

### Strengths and limitations of study

Strengths of this systematic review include the following: an extensive search algorithm developed by an academic librarian with expertise in this field; duplicate assessment of citations, abstracts, and full texts; inclusion of several outcomes relevant to patients, physicians, and policy makers; and use of the GRADE approach to evaluate the evidence. Limitations of our review reflect problems with the individual studies that we included. First, the many differences in crowdsourced interventions and their measurement made it difficult to pool data. Second, given that crowdsourcing is an emergent approach to health problems, there were many potential search terms to identify crowdsourcing research studies. Third, few studies included data on cost and feasibility as outcomes. Fourth, the data included many observational studies and had other methodological limitations. Fifth, the large majority of studies were conducted in high-income countries, highlighting the need for greater research focused on LMIC settings.

In comparison with previous systematic reviews [18, 22], we included many more studies. This reflects the substantial growth in the field of crowdsourcing over the past several years. Our review helps to define this emerging approach, with greater rigor than earlier reviews. We included outcomes (cost, feasibility) that were not examined in other systematic reviews.

Evidence from 17 observational studies examining crowdsourcing to evaluate surgical skills suggests the usefulness of this approach. Evaluating surgical skill is critical for surgeons at all levels of training. However, surgical skill evaluation can take months when relying on video assessment from qualified surgeons [63]. A crowdsourcing approach could increase the efficiency, timeliness, and thoroughness of feedback [33]. Crowdsourcing is now routinely used for surgical skill evaluation by the American Urological Association, BlueCross BlueShield, and over twenty major medical centers [64]. A potential limitation of the evidence is that the data to support this approach have come exclusively from high-income countries. Further research on crowdsourcing for surgical skill evaluation in low- and middle-income countries is needed.

Data from seven studies, including two RCTs, also suggest that crowdsourcing is an effective and cost-saving method for creating sexual health messages. The utility of crowdsourcing in this field may be related to the extent to which social and behavioral norms influence the effectiveness of sexual health interventions. The extensive community engagement involved in crowdsourcing may help to improve the acceptability of the intervention among key affected populations by drawing directly upon community member perspectives [45, 46, 48]. Based on the evidence that crowdsourcing approaches can effectively promote sexual health, several local, regional and global policy-makers have recommended this practice [10, 65]. The UNICEF/UNDP/World Bank/WHO Special Programme for Research and Training in Tropical Diseases has used crowdsourcing in several projects [21, 66].

Six studies evaluated layperson facilitated out-of-hospital CPR. These included two RCTs and four observational studies, all conducted in HICs, which indicate that crowdsourcing approaches to out-of-hospital CPR may increase CPR initiation, but may not decrease the time to CPR initiation. A scientific statement from the American Heart Association identified crowdsourcing approaches to increase out-of-hospital CPR as a priority area [67]. These approaches require telecommunication infrastructure and emergency medical services that make LMIC implementation more difficult, although increased smart phone penetration present an opportunity for user-friendly apps.

We also found that crowdsourcing may be useful in the development of artificial intelligence projects. Four studies annotated medical data in order to train machine learning algorithms [53, 55–57]. Especially as crowdsourcing solicits input from large numbers of people, the resulting big data may provide a platform for machine learning. In addition, one open challenge was able to effecively develop a machine learning algorithm [54].

Our systematic review has implications for applying crowdsourcing approaches to inform health policy and research. From a policy perspective, the diverse LMIC settings and relatively low cost in the six sexual health message studies suggest that crowdsourcing for developing sexual health messages may be useful in other LMICs. A crowdsourcing approach could also be useful to inform the development of public health policy, for example, by developing strategies to scale-up hepatitis testing and improve service delivery [68]. From a research perspective, the lack of robust studies suggests the need for more randomized controlled trials with clinical outcomes. This is a major gap in the literature that requires attention. One example of an effective use of crowdsourcing in an RCT design includes a recently completed large-scale, eight-city study of crowdsourcing to promote HIV testing [18], which demonstrated the value of crowdsourcing for enhancing public health campaigns. This systematic review data can be used to refine and standardize crowdsourcing approaches for specific healthcare contexts.

This systematic review collected evidence from a broad range of topics in health and medicine where crowdsourcing has been implemented and evaluated. Crowdsourcing breaks new ground in health and medical research, introducing the potential for mass community engagement and community-driven interventions.

## Conclusions

This systematic review found a wide range of evidence supporting the use of crowdsourcing in medicine. We found more robust research studies evaluating surgical skills, organizing out-of-hospital layperson CPR, and creating sexual health messages. These studies demonstrate a growing base of evidence to inform the use of crowdsourcing in artificial intelligence and related medical research. In addition, these studies suggest that crowdsourcing can broaden public engagement in medical research because members of the public can submit ideas, judge submissions, and serve on organizing committees. Further implementation and evaluation of crowdsourcing approaches are warranted.

## Supplementary information

**Additional file 1: Tables S1-S7.** Search algorithms for PubMed, Embase, CINAHL, Web of Science, PsychInfo, Cochrane Library, and ABI/Inform.

**Additional file 2: Table S8.** Bias assessment of 17 studies examining a crowdsourcing approach to surgical technique evaluation.

**Additional file 3: Table S9.** GRADE evidence profile for assessment of surgical performance.

**Additional file 4: Figure S10.** Forrest plots for pooled RCT data examining smoking cessation studies (top panel), depression studies (middle panel), and sexual health studies (bottom panel).

**Additional file 5: Table S11.** Bias assessment of four non-RCT studies evaluating innovation design contests to develop sexual health messages.

**Additional file 6: Table S12.** Bias assessment of two RCT studies evaluating innovation design contests to develop sexual health messages.

**Additional file 7: Table S13.** GRADE evidence profile for studies evaluating innovation design contests to develop sexual health messages.

**Additional file 8: Table S14.** Bias assessment of non-RCT studies exploring out-of-hospital CPR.

**Additional file 9: Table S15.** Bias assessment of RCT studies exploring out-of-hospital CPR.

**Additional file 10: Table S16.** GRADE evidence profile for studies exploring out-of-hospital CPR.

## Data Availability

The datasets used and/or analyzed during the current study are available from the corresponding author on reasonable request.

## Abbreviations

CPR: Cardiopulmonary resuscitation
HIV: Human immunodeficiency virus
LMICs: Low and middle income countries
PRISMA: Preferred Reporting Items for Systematic Reviews and Meta-Analyses
RCT: Randomized controlled trial
