# Supplementary material for: Crowdsourcing in health and medical research: a systematic review
Source: Infect Dis Poverty. 2020 Jan 20;9:8. doi: 10.1186/s40249-020-0622-9 (PMC6971908; doi:10.1186/s40249-020-0622-9)
Supplement: Supplementary file 1 — Additional file 1: Tables S1-S7. Search algorithms for PubMed, Embase, CINAHL, Web of Science, PsychInfo, Cochrane Library, and ABI/Inform. [file 40249_2020_622_MOESM1_ESM.docx]

**Additional File 1.** **Tables S1-S7. Search algorithms for PubMed, Embase, CINAHL, Web of Science, PsychInfo, Cochrane Library, and ABI/Inform.**

| **PubMed – 9.4.2019** | | |
| --- | --- | --- |
| Search # | Query | Results |
| #1 | (crowdsourcing[mesh] OR crowdsourcing[tiab] OR crowd sourced[tw] OR crowdsource[tw] OR crowd source[tw] OR crowdsourced[tw] OR competitive behavior[tiab] OR collective intelligence[tw] OR collective wisdom[tw] OR crowd science[tw] OR citizen science[tw]) | 2686 |
| #2 | (Global Health[MESH] OR Global Health[TIAB] OR Public Health[Mesh] OR Public Health[TIAB] OR Community Health[tw] OR Health Behavior[MESH] OR Health Behavior[TIAB] OR Health Behaviour[TIAB] OR Health Promotion[MESH] OR Health Promotion[TIAB] OR Disease Prevention[TIAB] OR Health Message[TIAB] OR Prevention[TIAB] OR "Health Knowledge, Attitudes, Practice"[Mesh] OR Medicine[TIAB] OR Medical[TIAB] OR Patients[TIAB] OR doctor[tiab] OR Doctors[TIAB] OR physician[tiab] OR Physicians[TIAB] OR Clinical[TIAB] OR healthcare[tw] OR health care[tw]) | 12588449 |
| #3 | #1 AND #2 | 1289 |

| **Embase – 9.4.2019** | | |
| --- | --- | --- |
| Search # | Query | Results |
| #1 | (‘crowdsourcing’/exp OR (crowdsourcing OR ‘crowd sourcing’ OR ‘crowd sourced’ OR crowdsourced OR ‘crowd source’ OR crowdsource OR ‘competitive behavior’ OR ‘collective intelligence’ OR ‘collective wisdom’ OR ‘crowd science’ OR ‘citizen science’):ab,ti) | 2,756 |
| #2 | (‘health’/exp OR ‘public health’/exp OR ‘health behavior’/exp OR ‘health promotion’/exp OR (health OR ‘global health’ OR ‘public health’ OR ‘community health’ OR ‘health behavior’ OR ‘health behaviour’ OR ‘health promotion’ OR ‘disease prevention’ OR medicine OR medical OR patients OR doctor OR doctors OR physician OR physicians OR clinical OR healthcare OR ‘health care’):ab,ti) | 12,840,653 |
| #3 | #1 AND #2 | 1,155 |

| **CINAHL – 9.4.2019** | | |
| --- | --- | --- |
| Search # | Query | Results |
| #1 | TI ( crowdsourcing OR "crowd sourced" OR crowdsource OR "crowd source" OR crowdsourced OR "competitive behavior" OR "collective intelligence" OR "collective wisdom" OR "crowd science" OR "citizen science" OR "citizen scientist" OR "citizen scientists" ) OR AB ( crowdsourcing OR "crowd sourced" OR crowdsource OR "crowd source" OR crowdsourced OR "competitive behavior" OR "collective intelligence" OR "collective wisdom" OR "crowd science" OR "citizen science" OR "citizen scientist" OR "citizen scientists" ) OR MH crowdsourcing | 884 |
| #2 | MH ( "research, medical" OR "world health" OR "public health" OR "health behavior" OR "health promotion" ) OR TI ( “world health” OR “global health” OR “public health” OR “community health” OR “health behavior” OR “health behaviour” OR “health promotion” OR “disease prevention” OR medicine OR medical OR patients OR doctor OR doctors OR physician OR physicians OR clinical OR healthcare OR “health care” ) OR AB ( “world health” OR “global health” OR “public health” OR “community health” OR “health behavior” OR “health behaviour” OR “health promotion” OR “disease prevention” OR medicine OR medical OR patients OR doctor OR doctors OR physician OR physicians OR clinical OR healthcare OR “health care” ) | 2,254,327 |
| #3 | S1 AND S2 | 358 |

| **Web of Science – 9.4.2019** | | |
| --- | --- | --- |
| Search # | Query | Results |
| #1 | crowdsourcing OR "crowd sourced" OR crowdsource OR "crowd source" OR crowdsourced OR "competitive behavior" OR "collective intelligence" OR "collective wisdom" OR "crowd science" OR "citizen science" OR "citizen scientist" OR "citizen scientists" | 15,485 |
| #2 | “world health” OR “global health” OR “public health” OR “community health” OR “health behavior” OR “health behaviour” OR “health promotion” OR “disease prevention” OR medicine OR medical OR patients OR doctor OR doctors OR physician OR physicians OR clinical OR healthcare OR “health care” | 8,557,674 |
| #3 | #1 AND #2 | 969 |

| **PsycINFO – 9.4.2019** | | |
| --- | --- | --- |
| Search # | Query | Results |
| #1 | DE "Crowdsourcing" OR TI (crowdsourcing OR "crowd sourced" OR crowdsource OR "crowd source" OR crowdsourced OR "competitive behavior" OR "collective intelligence" OR "collective wisdom" OR "crowd science" OR "citizen science" OR "citizen scientist" OR "citizen scientists") OR AB (crowdsourcing OR "crowd sourced" OR crowdsource OR "crowd source" OR crowdsourced OR "competitive behavior" OR "collective intelligence" OR "collective wisdom" OR "crowd science" OR "citizen science" OR "citizen scientist" OR "citizen scientists") | 1,630 |
| #2 | DE ( "global health" OR "public health" OR "health behavior" OR "health promotion" ) OR TI ( “world health” OR “global health” OR “public health” OR “community health” OR “health behavior” OR “health behaviour” OR “health promotion” OR “disease prevention” OR medicine OR medical OR patients OR doctor OR doctors OR physician OR physicians OR clinical OR healthcare OR “health care” ) OR AB ( “world health” OR “global health” OR “public health” OR “community health” OR “health behavior” OR “health behaviour” OR “health promotion” OR “disease prevention” OR medicine OR medical OR patients OR doctor OR doctors OR physician OR physicians OR clinical OR healthcare OR “health care” ) | 1,197,450 |
| #3 | S1 AND S2 | 219 |

| **Cochrane Library – 9.4.2019** | | |
| --- | --- | --- |
| Search # | Query | Results |
| #1 | crowdsourcing or "crowd sourced" or crowdsource or "crowd source" or crowdsourced or "competitive behavior" or "collective intelligence" or "collective wisdom" or "crowd science" or "citizen science" or "citizen scientist" or "citizen scientists":ti,ab,kw (Word variations have been searched) | 337 |
| #2 | "world health" or "global health" or "public health" or "community health" or "health behavior" or "health behaviour" or "health promotion" or "disease prevention" or medicine or medical or patients or doctor or doctors or physician or physicians or clinical or healthcare or "health care":ti,ab,kw (Word variations have been searched) | 1347017 |
| #3 | #1 AND #2 | 211 |

| **ABI/Inform – 9.4.2019** | | |
| --- | --- | --- |
| Search # | Query | Results |
| #1 | Noft(crowdsourcing or "crowd sourced" or crowdsource or "crowd source" or crowdsourced or "competitive behavior" or "collective intelligence" or "collective wisdom" or "crowd science" or "citizen science" or "citizen scientist" or "citizen scientists") | 11,887 |
| #2 | Noft("world health" or "global health" or "public health" or "community health" or "health behavior" or "health behaviour" or "health promotion" or "disease prevention" or medicine or medical or patients or doctor or doctors or physician or physicians or clinical or healthcare or "health care") | 7,956,612 |
| #3 | #1 AND #2 | 742 |
| #4 | #1 AND #2 NOT (wire feeds AND magazines AND newspapers AND blogs, podcasts & websites) | 343 |
