# Supplementary material for: Crowdsourcing in health and medical research: a systematic review
Source: Infect Dis Poverty. 2020 Jan 20;9:8. doi: 10.1186/s40249-020-0622-9 (PMC6971908; doi:10.1186/s40249-020-0622-9)
Supplement: Supplementary file 2 — Additional file 2: Table S8. Bias assessment of 17 studies examining a crowdsourcing approach to surgical technique evaluation. [file 40249_2020_622_MOESM2_ESM.docx]

**Additional File 2: Table S8. Bias assessment of 17 studies examining a crowdsourcing approach to surgical technique evaluation.**

| Study | Year | Design | Total experts | Total laypersons | Industry funding | Eligibility criteria | Confounding | Exposure/  Outcome | Follow-up |
| --- | --- | --- | --- | --- | --- | --- | --- | --- | --- |
| Aghdasi | 2015 | Survey | 3 | 30 | T.S.L. is equity owner of CSATS, Inc. | Appropriate: expert surgeons, Mechanical Turk workers | Medium: large differential in time to receive full responses | Medium: 2 additional surveys to laypersons, who displayed greater variation | Medium: complete but very small sample size |
| Chen | 2014 | Survey | 10 | 611 | None | Appropriate: teaching surgeons, Mechanical Turk workers, Facebook users | Medium: differential time to receive full responses | Medium: only a single video was assessed | Medium: 9 (90%) for surgeons, 409 (82%) for Mechanical Turk, 67 (63%) for Facebook |
| Deal | 2016 | Survey: GOALS validated rating instrument | 6 | 203 | None | Mechanical Turk novice learners only | High: low inter-rater reliability | Low: minimum of 30 evaluations on each of the same 21 video clips | Low: time to receiving final scores relatively short |
| Ghani | 2016 | Survey: Global skill (GEARS) and procedure-specific skill (RACE) | 25 | 680 | Blue Cross Blue Shield of Michigan, C-SATS, Inc | Appropriate: surgeons and Mechanical Turk workers | Low: fitted linear mixed-effects models | Low: 76 video clips | Low: time to receiving final scores 15d for surgeons, 38h for crowd |
| Holst (dry-lab robotic suturing) | 2015 | Survey: GEARS | 3 | 250 | CSATS, Inc. | Appropriate: surgeons and Mechanical Turk workers | Low: high IRR of 0.91 | Medium: 5 videos | Low: not a problem |
| Holst (urinary bladder) | 2015 | Survey: GEARS | 7 | 600 | CSATS, Inc. | Appropriate: surgeons and Mechanical Turk workers | Low: high inter-rater reliability of surgeons at 0.89 | Medium: 12 videos | Low: not a problem |
| Kowalewski | 2016 | Survey: GOALS | 5 | 1840 ratings | None | Appropriate: faculty experts and Mechanical Turk workers | Medium: high IRR for faculty, lower for crowd (0.79) for pegboard tasks | Low: 24 videos | Medium: a sizeable number of crowd worker reviews were discarded |
| Maier-Hein | 2014 | Evaluation of object classifier depending on group | Not given | Not given | None | Appropriate: Experts and Mechanical Turk workers | Low: identical testing images for all methods | Medium: 6 videos; multiple linear regressions | Low: not a problem |
| Malpani | 2015 | Survey of existing surgical training task segments | 8 | 147 | None | Medium: study call was open to all within Johns Hopkins community (crowd) | High: low IRR within the crowd (0.41) and experts (0.55) | Low: pairwise rankings | Low: not a problem |
| Goldenberg | 2017 | Survey: GEARS | 5 | 2,142 ratings | None reported | Medium: Mechanical Turk inclusion criteria not detailed | High: low internal consistency across expert ratings (ICC=-0.108) | Low: 18 videos, raters were blinded to outcomes | Low: not a problem |
| Hu | 2017 | Survey: Objective Structured Assessments of Technical Skills (OSATS) | 3 | 40 | None reported | Appropriate: Experts and Mechanical Turk workers who passed screening process | Medium, IRR among experts = 0.72-0.88 | Low: 15 study videos | Low: not a problem |
| Lee | 2017 | Survey: GEARS | 2 | Not given | None | Medium: Mechanical Turk inclusion criteria not detailed | Low: high internal consistency across expert ratings (ICC =0.877) | Low: 4 tasks each for 105 participants | Low: not a problem |
| Peabody | 2015 | Survey: GEARS, RACE | 318 ratings (25 surgical experts) | 2,531 ratings | None | Appropriate: Surgical experts and Mechanical Turk workers using CSATS | High: expert GEARS ratings lacked internal consistency (ICC=0.24) | Low: linear mixed effects models | Low: not a problem |
| Polin | 2016 | Surgey: Robotic-Objective Structured Assessments of Technical Skills (R-OSATS) | 448 | Not given (previously rated) | Medtronic, Intuitive Surgical, C-SATS | Appropriate: Mechanical Turk workers needed >95% approval or higher from previous assignments | Medium: existing expert evaluator R-OSATS scores had moderate IRR (0.55-0.69). | Low: 60 videos | Low: not a problem |
| Powers | 2016 | Survey: GEARS assessed through CSATS | Not given | 548 ratings | None | Appropriate: Mechanical Turk workers needed a 95% approval or higher, urologic surgeons needed a minimum of 500 robotic cases as primary surgeon | High: low internal consistency across content expert ratings (ICC=0.38) | Low: 14 video clips each received at least 3 expert and 30 Turk evaluations | Low: not a problem |
| Vernez | 2017 | Survey: OSATS, GEARS, GOALS | 150 ratings (6 experts) | 3,939 ratings overall for 4 tasks | None | Appropriate: Mechanical Turk workers needed a 95% approval or higher, min of 100 tasks, module | Medium: good IRR for experts (>0.70), not available for crowd | Low: 4 tasks for each of 25 students | Low: not a problem |
| White | 2014 | Survey: Global Assessment of Basic Surgical Skills (GABSS) | 3 | 1,000 ratings | None | Medium: Mechanical Turk inclusion criteria not detailed | Excellent IRR among surgeons (IRR=0.9) and among crowd (IRR=0.85) | Low: 10 videos were blinded for 3 different items | Low: not a problem |
