# Supplementary material for: Crowdsourcing in health and medical research: a systematic review
Source: Infect Dis Poverty. 2020 Jan 20;9:8. doi: 10.1186/s40249-020-0622-9 (PMC6971908; doi:10.1186/s40249-020-0622-9)
Supplement: Supplementary file 3 — Additional file 3: Table S9. GRADE evidence profile for assessment of surgical performance. [file 40249_2020_622_MOESM3_ESM.docx]

**Additional File 3: Table S9. GRADE evidence profile for assessment of surgical performance.**

| Quality assessment | | | | | | | Agreement | | Quality | Importance |
| --- | --- | --- | --- | --- | --- | --- | --- | --- | --- | --- |
| No. of studies | Study design | Risk of bias | Consistency | Directness | Precision | Other considerations | Cronbach’s alpha | Pearson’s correlation coefficient |  |  |
| Evaluation of surgical performance (Aghdasi 2015; Chen 2014; Deal 2016; Ghani 2016; Goldenberg 2017; Holst 2015; Holst 2015; Hu 2017; Kowalewski 2016; Lee 2017; Malpani 2015; Peabody 2015; Polin 2016; Powers 2016; Vernez 2017; White 2014 | | | | | | | | | | |
| 13 | Cross-sectional observational | Not serious | Not serious | No serious indirectness | Mild imprecision | Publication bias limited | Median: 0.59 (0.53 – 0.65) | Median: 0.76 (0.74 – 0.77) | 2/4 (Low) | Important |
| Quality assessment | | | | | | | Effect | | Quality | Importance |
| No. of studies | Study design | Risk of bias | Consistency | Directness | Precision | Other considerations | Median time (range) | Median difference between crowd and experts (range)* |  |  |
| Speed of ratings (Aghdasi 2015; Chen 2014; Deal 2016; Ghani 2016; Holst 2015; Holst 2015; Kowalewski 2016;Maier-Hein 2014; Malpani 2015; Peabody 2015; Powers 2016; Vernez 2017; White 2014) | | | | | | | | | | |
| 14 | Cross-sectional observational | Not serious | Not serious | No serious indirectness | Precise | None | Crowd: 18hrs (3hrs-48hrs); Experts: 31 days (10 days- 120 days) | 19 days (9 days-118 days) | 3/4 (Medium) | Important |

*For each study, the difference in rating time was taken between the crowd and experts. From that set of times, the median and range are reported.
