# Supplementary material for: Crowdsourcing in health and medical research: a systematic review
Source: Infect Dis Poverty. 2020 Jan 20;9:8. doi: 10.1186/s40249-020-0622-9 (PMC6971908; doi:10.1186/s40249-020-0622-9)

**Additional file 4: Figure S10. Forest plots for pooled RCT data examining smoking cessation studies (top panel), depression studies (middle panel), and sexual health studies (bottom panel).**


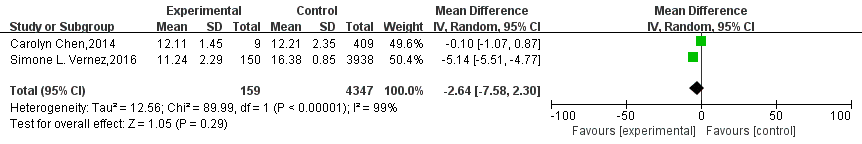

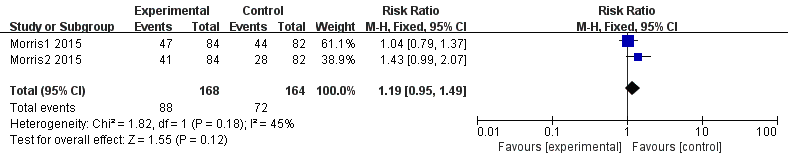

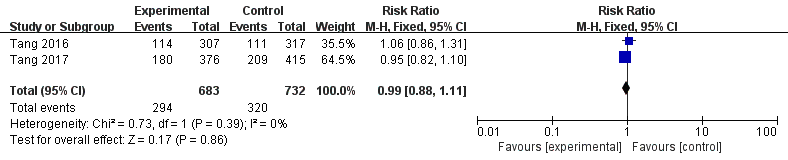

Supplement: Supplementary file 4 — Additional file 4: Figure S10. Forrest plots for pooled RCT data examining smoking cessation studies (top panel), depression studies (middle panel), and sexual health studies (bottom panel). [file 40249_2020_622_MOESM4_ESM.docx]
