# Supplementary material for: Crowdsourcing in health and medical research: a systematic review
Source: Infect Dis Poverty. 2020 Jan 20;9:8. doi: 10.1186/s40249-020-0622-9 (PMC6971908; doi:10.1186/s40249-020-0622-9)
Supplement: Supplementary file 5 — Additional file 5: Table S11. Bias assessment of four non-RCT studies evaluating innovation design contests to develop sexual health messages. [file 40249_2020_622_MOESM5_ESM.docx]

**Additional File 5: Table S11. Bias assessment of four non-RCT studies evaluating innovation design contests to develop sexual health messages.**

| Study | Year | Design | Total participants | Population | Industry funding | Eligibility criteria | Confounding | Exposure/  Outcome | Follow-up |
| --- | --- | --- | --- | --- | --- | --- | --- | --- | --- |
| Beres | 2013 | Observational study | 586 | Males and females 10-24 years old from 6 sub-Saharan African countries | None | Low: stratified random sample of narratives | Low: countries selected based on diverse HIV prevalence rates | Medium: variable exposure to risky behaviors and HIV testing | Unclear |
| Catallozzi | 2013 | Observational study | 69 videos | Entire Australian public | Novartis Pharmaceuticals | Low: open to all Australians | Low: Content diverse | Medium: effectiveness of videos not assessed | 63 (91%) were evaluated |
| Keller | 1997 | Observational study | 1,681 | Men and women ages 15-19 in Kampala, Uganda | None | Low: judges were the target audience | Low: large sample size | High: no clear comparator group | Unclear |
| Zhang | 2015 | Observational study | 103 (7+96 entries) | CBOs delivering HIV testing in Greater China; people living in China over 30 years of age | None | Low: Clear criteria | Medium: small sample size, fewer controls | Low: same exposures and outcomes | Low: not a problem |
