# Supplementary material for: Crowdsourcing in health and medical research: a systematic review
Source: Infect Dis Poverty. 2020 Jan 20;9:8. doi: 10.1186/s40249-020-0622-9 (PMC6971908; doi:10.1186/s40249-020-0622-9)
Supplement: Supplementary file 6 — Additional file 6: Table S12. Bias assessment of two RCT studies evaluating innovation design contests to develop sexual health messages. [file 40249_2020_622_MOESM6_ESM.docx]

**Additional File 6. Table S12. Bias assessment of 2 RCT studies evaluating innovation design contests to develop sexual health messages.**

| Study | Year | Design | Total participants | Population | Industry funding | Allocation: generation | Allocation: concealment | Blinding: participants | Blinding: assessors | Outcome: complete | Outcome: selective | Other bias |
| --- | --- | --- | --- | --- | --- | --- | --- | --- | --- | --- | --- | --- |
| Tang | 2019 | Non-inferior RCT | 1173 | Chinese MSM >16 years old, had condomless anal sex | None | Low | Low | Medium: potential for multiple video assessments | Low | Medium: attrition 33% by 3-months follow-up | Low | Low |
| Tang | 2018 | Stepped wedge RCT | 1381 | Chinese MSM and transgender individuals > 16 years old, not HIV tested in past 3 months |  | Low | Low | Low | Low | Low | Low | Low |
