# Supplementary material for: Crowdsourcing in health and medical research: a systematic review
Source: Infect Dis Poverty. 2020 Jan 20;9:8. doi: 10.1186/s40249-020-0622-9 (PMC6971908; doi:10.1186/s40249-020-0622-9)
Supplement: Supplementary file 7 — Additional file 7: Table S13. GRADE evidence profile for studies evaluating innovation design contests to develop sexual health messages. [file 40249_2020_622_MOESM7_ESM.docx]

**Additional File 7. Table S13. GRADE evidence profile for studies evaluating innovation design contests to develop sexual health messages.**

| Quality assessment | | | | | | | Effect | Quality | Importance |
| --- | --- | --- | --- | --- | --- | --- | --- | --- | --- |
| No. of studies | Study design | Risk of bias | Consistency | Directness | Precision | Other considerations | Relative difference (95% CI) |  |  |
| Increased HIV testing uptake (Beres 2013; Tang 2019; Zhang 2015; Tang 2018) | | | | | | | | | |
| 4 | 2 RCTs, 2 observational studies | Not serious | Not serious | Some indirectness | Mild imprecision | Not serious | 2.1% (-5.4%, 9.7%) in favor of crowdsourced intervention | 3/4 (Medium) | Critical |
| Increased communication about sexual health among youth (Beres 2013; Catallozzi 2013; Keller 1997; Tang 2019; Zhang 2015; Tang 2018) | | | | | | | | | |
| 6 | 2 RCTs, 4 observational studies | Mild | Not serious | Some indirectness | Mild imprecision | Magnitude of effects not large | 1.3% difference (-4.8%, 7.4%) in favor of crowdsourced intervention | 3/4 (Medium) | Important |
