# Supplementary material for: Crowdsourcing in health and medical research: a systematic review
Source: Infect Dis Poverty. 2020 Jan 20;9:8. doi: 10.1186/s40249-020-0622-9 (PMC6971908; doi:10.1186/s40249-020-0622-9)
Supplement: Supplementary file 8 — Additional file 8: Table S14. Bias assessment of non-RCT studies exploring out-of-hospital CPR. [file 40249_2020_622_MOESM8_ESM.docx]

**Additional File 8. Table S14. Bias assessment of non-RCT studies exploring out-of-hospital CPR.**

| Study | Year | Design | Total participants | Population | Industry funding | Eligibility criteria | Confounding | Exposure/  Outcome | Follow-up |
| --- | --- | --- | --- | --- | --- | --- | --- | --- | --- |
| Brooks | 2013 | Observational study | 41,885 downloads | Community members across California, Kentucky, and South Dakota | None | Low: not a problem | Medium: distance to location | Low: not problematic | Medium: registering device does not equate to receiving alerts |
| Narikawa | 2014 | Prospective study | 1,360 patient care records | Trained laypersons in Tonchigi prefecture, Japan | None | Low: not a problem | Low | Medium: CFR protocols differ slightly | Low: not a problem |
| Ringh | 2011 | Observational study | 1,271-1,801 | Mobile responders trained in CPR in Stockholm, Sweden | LEKAB Communication Systems | Low: appropriate | Low | Medium: dual dispatch system including police and fire department not compared | Low: not a problem |
| Scholten | 2011 | Cross-sectional survey | 2168 | Laypersons in Twente, Netherlands with email and SMS | None | Low: clear criteria | Low | Low: not problematic | Medium: loss to follow-up in survey |
