# Supplementary material for: Crowdsourcing in health and medical research: a systematic review
Source: Infect Dis Poverty. 2020 Jan 20;9:8. doi: 10.1186/s40249-020-0622-9 (PMC6971908; doi:10.1186/s40249-020-0622-9)
Supplement: Supplementary file 9 — Additional file 9: Table S15. Bias assessment of RCT studies exploring out-of-hospital CPR. [file 40249_2020_622_MOESM9_ESM.docx]

**Additional File 9. Table S15. Bias assessment of RCT studies exploring out-of-hospital CPR.**

| Study | Year | Design | Total participants | Population | Industry funding | Allocation: generation | Allocation: concealment | Blinding: participants | Blinding: assessors | Outcome: complete | Outcome: selective | Other bias |
| --- | --- | --- | --- | --- | --- | --- | --- | --- | --- | --- | --- | --- |
| Ringh | 2015 | RCT | 665 (305 in intervention, 360 in control) | Lay volunteers trained in CPR in Sweden | None | Low | Low | Low | Low | Medium: mobile phone positioning system not used at night or in select cases | Low | Medium: single center, not powered for survival |
| Zanner | 2007 | RCT | 119 (55 in test, 64 in control) | Lay volunteer in Munich, Germany, majority high school students | Bitos and GmbH provided mobile phones running software | Low | Low | High: twice the percentage with no first aid knowledge in test group | Low | Medium: attrition 33% by 3-months follow-up | Low | Medium: small sample size |
