# Supplementary material for: Crowdsourcing in health and medical research: a systematic review
Source: Infect Dis Poverty. 2020 Jan 20;9:8. doi: 10.1186/s40249-020-0622-9 (PMC6971908; doi:10.1186/s40249-020-0622-9)
Supplement: Supplementary file 10 — Additional file 10: Table S16. GRADE evidence profile for studies exploring out-of-hospital CPR. [file 40249_2020_622_MOESM10_ESM.docx]

**Additional File 10. Table S16. GRADE evidence profile for studies exploring out-of-hospital CPR**

| Quality assessment | | | | | | | Effect | Quality | Importance |
| --- | --- | --- | --- | --- | --- | --- | --- | --- | --- |
| No. of studies | Study design | Risk of bias | Consistency | Directness | Precision | Other considerations |  |  |  |
| Use of smartphone apps or SMS to increase bystander-initiated CPR (Narikawa 2014; Ringh 2015; Zanner 2007) | | | | | | | | | |
| 3 | 2 RCTs, one observational study | Not serious | Mild inconsistency | No serious indirectness | No serious imprecision | Not serious | 14 percentage points higher in test group (95% CI 6 to 21, p<0.001); higher score by test group but not significant (21.1 vs. 19.9, p=0.30); comparable sensitivity and specificity | 3/4 (Medium) | Critical |
| Response time of bystanders compared to traditional methods (Ringh 2011; Scholten 2011; Zanner 2007) | | | | | | | | | |
| 3 | 2 RCTs, one observational study | Mild | Mild inconsistent* | No serious indirectness | Mild imprecision | Effective blinding of concern | Inconclusive (lay persons not necessarily faster than EMS) | 2/4 (Low) | Critical |

*Two studies (Ringh 2011, Scholten 2011) both showed improved response time in the crowdsourced intervention compared to traditional methods. The Zanner 2007 study found the opposite. However, the traditional method group had higher baseline knowledge of CPR compared to the crowdsourced group.
